# How does power shape district health management team responsiveness to public feedback in low- and middle-income countries: an interpretive synthesis

**DOI:** 10.1093/heapol/czac105

**Published:** 2022-12-06

**Authors:** Nancy Kagwanja, Sassy Molyneux, Eleanor Whyle, Benjamin Tsofa, Lucy Gilson

**Affiliations:** Health Systems Research Ethics Department, KEMRI-Wellcome Trust Research Programme, Hospital Road, P.O BOX 230-80108, Kilifi, Kenya; Health Systems Research Ethics Department, KEMRI-Wellcome Trust Research Programme, Hospital Road, P.O BOX 230-80108, Kilifi, Kenya; Centre for Tropical Medicine and Global Health, Nuffield Department of Medicine, University of Oxford, Old Road, Oxford OX3 7BN, UK; Health Systems and Policy Division, School of Public Health and Family Medicine, University of Cape Town, Falmouth Road, Observatory, Cape Town 7925, South Africa; Health Systems Research Ethics Department, KEMRI-Wellcome Trust Research Programme, Hospital Road, P.O BOX 230-80108, Kilifi, Kenya; Health Systems and Policy Division, School of Public Health and Family Medicine, University of Cape Town, Falmouth Road, Observatory, Cape Town 7925, South Africa; Department of Global Health and Development, London School of Hygiene and Tropical Medicine, Keppel Street, London WC1E 7HT, UK

**Keywords:** Health system, responsiveness, public, feedback, district health manager, district health management team, power

## Abstract

Responsiveness is a core element of World Health Organization’s health system framework, considered important for ensuring inclusive and accountable health systems. System-wide responsiveness requires system-wide action, and district health management teams (DHMTs) play critical governance roles in many health systems. However, there is little evidence on how DHMTs enhance health system responsiveness. We conducted this interpretive literature review to understand how DHMTs receive and respond to public feedback and how power influences these processes. A better understanding of power dynamics could strengthen responsiveness and improve health system performance. Our interpretive synthesis drew on English language articles published between 2000 and 2021. Our search in PubMed, Google Scholar and Scopus combined terms related to responsiveness (feedback and accountability) and DHMTs (district health manager) yielding 703 articles. We retained 21 articles after screening. We applied Gaventa’s power cube and Long’s actor interface frameworks to synthesize insights about power. Our analysis identified complex power practices across a range of interfaces involving the public, health system and political actors. Power dynamics were rooted in social and organizational power relationships, personal characteristics (interests, attitudes and previous experiences) and world-views (values and beliefs). DHMTs’ exercise of ‘visible power’ sometimes supported responsiveness; however, they were undermined by the ‘invisible power’ of public sector bureaucracy that shaped generation of responses. Invisible power, manifesting in the subconscious influence of historical marginalization, patriarchal norms and poverty, hindered vulnerable groups from providing feedback. We also identified ‘hidden power’ as influencing what feedback DHMTs received and from whom. Our work highlights the influence of social norms, structures and discrimination on power distribution among actors interacting with, and within, the DHMT. Responsiveness can be strengthened by recognising and building on actors' life-worlds (lived experiences) while paying attention to the broader context in which these life-worlds are embedded.

Key messages

- Applying actor interface analysis and Gaventa’s power cube can help health policy analysts examine the interactions between structural influences and actor agency. This has value in understanding implementation challenges and in drawing out different dimensions of a goal as complex as health system responsiveness.
- In a health system decision-making space such as the DHMT, power can be wielded in both positive and negative ways. How this power is exercised has a reinforcing effect on the public’s sharing of feedback. Positive power practices support the generation of responses and even more feedback from the public. Negative power practices can limit generation of responses and the public’s sharing of feedback.
- Responsiveness could be strengthened by recognizing and building on the actor life-worlds that influence responsiveness practice. This could include leveraging politicians’ power and personal interests while strengthening feedback channels to ensure meaningful public involvement and inclusivity and interventions to shape DHMTs’ world-views and work environments to support responsiveness to public feedback.
- Further research about power in the practice of health system responsiveness could test the conclusions and conceptual framework generated by this synthesis, in DHMTs in other contexts and in other spaces within the health system where decision-making on public feedback occurs.

## Introduction

Responsiveness is one of the three health system goals, alongside health outcomes and fairness in financing introduced by the World Health Report of 2000 (WHO, 2000). Health system responsiveness has been judged necessary to provide inclusive, participatory and accountable services (Rottger *et al.*, 2015; Askari *et al.*, 2016). However, there is evidence that the public experiences difficulty in engaging with and eliciting responses from the health system (Golooba‐Mutebi, 2005; Gurung *et al.*, 2017). Furthermore, responsiveness is intended to draw attention to the needs of minority groups, but ‘inequalities in responsiveness have received little attention’ (Jones *et al.*, 2011). While multiple public feedback mechanisms have the potential to enhance health system responsiveness (Molyneux *et al.*, 2012; Cleary *et al.*, 2017), there is limited information on their functioning and success in building system-level responsiveness—rather than on individual feedback pathways (Lodenstein *et al.*, 2017; Whyle and Olivier, 2017).

This article presents an interpretive synthesis (Pope *et al.*, 2007; Gilson, 2014) that addresses the overarching question: how do subnational health management teams receive, process and respond to public feedback? We sought first to identify whether and through what channels subnational health managers receive feedback from the public, how this feedback is analysed and whether responses to this feedback are generated and shared with the public. Second, because power has been cited as an influence on the responsiveness of health system agents (Berlan and Shiffman, 2012; Lodenstein *et al.*, 2017), we included sub-questions related to power dynamics at the subnational level. We sought to understand how actors exercise power when receiving and responding to public feedback at the subnational level, why actors exercise power and what the effects of their power practices are. Research synthesis has a value in answering policy questions related to service delivery and organizational- and system-level change (Pope *et al.*, 2007). As an interpretive synthesis, this article aims to draw out an understanding from the existing literature of whether and how power shapes responsiveness to public feedback at the subnational level and to consider what strategies might be deployed to deepen responsiveness. Synthesizing existing evidence also provides a platform for future empirical work to examine these issues more deeply (Gilson, 2014; Gilson *et al.*, 2014a). Thus, the third aim of this work was to present conceptual insights drawn from the synthesized articles that could inform policy and research on health system responsiveness. The findings of this paper would be potentially relevant to policymakers, regional and district health managers, researchers and non-governmental organizations (NGOs) with an interest in promoting the inclusion of public input in shaping health systems.

Our definition of health system responsiveness is how the health system reacts or responds to the public’s needs and concerns (Whyle and Olivier, 2017). We understand the following processes as constituting the ‘responsiveness pathway’ within health system decision-making: receiving, processing (could include analysis, integration and/or prioritization) and responding to feedback (Whyle and Olivier, 2017). In this article, we focus on subnational health management teams^1^, which might be referred to as district health management teams (DHMTs) or Sub-county Health Management Teams (SCHMTs) depending on the country. We consider these teams to be a processing space in the health system where feedback could be received and acted on. ‘Feedback’ refers to the views, concerns and information shared by the public; ‘feedback channel’ or ‘feedback mechanism’ refers to how information, views and concerns from the public reach DHMTs. Feedback mechanisms might be formal or informal. Formal mechanisms are those that are legislated or provided for in policy and include ‘community-level’ feedback mechanisms such as health facility committees (HFCs), intersectoral health forums or community monitoring (Molyneux *et al.*, 2012) and ‘individual-level’ feedback mechanisms such as suggestion boxes (Atela, 2013), exit surveys and incident reports (Khan *et al.*, 2021). Informal mechanisms are not necessarily mandated or legislated and might appear in contexts where formal mechanisms are absent or are considered ineffective by citizens (Tsai, 2007; Hossain, 2009; Lodenstein *et al.*, 2018). Informal mechanisms include individual complaints or compliments shared directly with frontline providers and health managers or via an intermediary and collective feedback such as public protests or ‘public buzz’ (conversations in public places) (Hossain, 2009; Lodenstein *et al.*, 2018).

There is increasing attention to the complex roles DHMTs play in managing and leading health systems at the district level in low- and middle-income countries (LMICs) (Kwamie *et al.*, 2015; Nyikuri *et al.*, 2017; Bulthuis *et al.*, 2021). However, there is little evidence about how public feedback is brought into DHMTs’ decision-making and of the influences on these processes. Although power is at the ‘heart of every policy process’ (p 361) (Erasmus and Gilson, 2008), including health system responsiveness (Berlan and Shiffman, 2012; Lodenstein *et al.*, 2017), there are few purposeful examinations of power in health policy and systems research (HPSR) (Gilson and Raphaely, 2008; Topp *et al.*, 2021) and even fewer examinations of power in the practice of health system responsiveness (Khan *et al.*, 2021). To strengthen responsiveness at the subnational level, a better understanding of how public feedback is handled within decision-making spaces such as the DHMT (including the influence of power) is important.

In the ‘Methods’ section, we describe the approach we adopted in conducting this work. We then present our findings in two parts: the first is a description of the various ways in which public feedback is received and responded to by DHMTs, and the second is a synthesis of the power dynamics influencing how DHMTs receive and generate responses to public feedback.

## Methods

We conducted a purposive review and interpretive synthesis (Thomas and Harden, 2008; Gilson, 2014). Interpretive synthesis allows researchers to draw conclusions on the collective meaning of pooled studies in a systematic manner (Gilson, 2014). This approach, also used more widely in HPSR (Erasmus, 2014; Gilson *et al.*, 2014b; Parashar *et al.*, 2020a), draws on studies that did not consider the review question and generates new interpretations of reported study experiences by going beyond the original studies during analysis (Pope *et al.*, 2007; Thomas and Harden, 2008). We have drawn on the enhancing transparency in reporting the synthesis of qualitative research guidelines (Tong *et al.*, 2012) and in reporting our synthesis methodology (see Supplementary Material 1).

### Data sources search strategy and screening

The search for papers was conducted on PubMed, Google Scholar and Scopus between December 2020 and March 2021 using the search criteria as presented in Table 1. The databases were chosen because they were free access and comprehensive and are known to cover health-related matters. A total of 694 papers were identified through database searches. NK made all the searches in consultation with a librarian. All the citations from the different databases were exported to Excel, and duplicates were removed. This was followed by screening of the title and abstracts for relevant papers (Supplementary Material 2). The eligibility of the studies selected was discussed with three members of the authorship team. Hand searching of the reference lists of articles identified was used to identify additional articles judged relevant to the review and synthesis questions. In total, 703 papers were identified.

**Table 1.** Search strategy

Term A^a^: Responsiveness Variants combined by OR	Subnational health management team Variants combined by OR
Responsive*, social accountability, accountability, community participation, community feedback, community participation, community voice, community engagement public feedback, public participation, stakeholder participation	District health management team*, sub-county health management team*, district health manager*, regional health management team*, regional health manager*, provincial health management team, provincial health manager*

a The two groups were ultimately combined with AND.

### Eligibility criteria and quality appraisal

**Figure 1.**
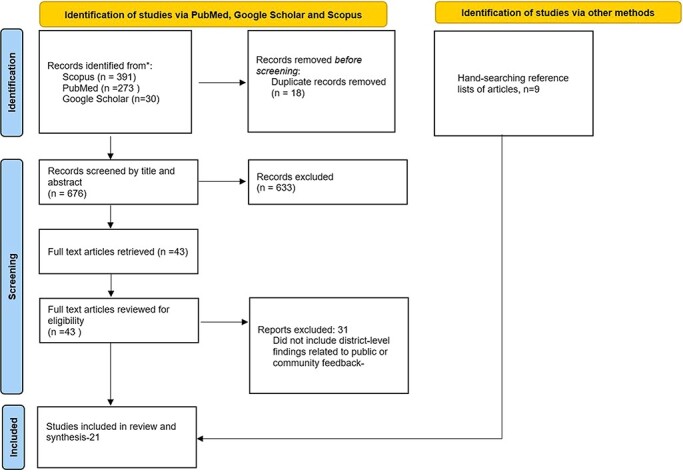
PRISMA flow diagram for screening of papers

Articles were included in this review if they met the following criteria: (1) they contained substantial content on DHMTs receiving, processing and/or responding to public input; (2) they focused on LMICs; (3) they were in English and (4) they were published between 2000 and 2021. The latter criterion was adopted because responsiveness was introduced as a health system goal by the World Health Organization (WHO) in 2000 (WHO, 2000). Twenty-one articles were retained after screening. Figure 1 summarizes the screening process. Selection of the articles included in the review combined assessment of specific relevance (empirical analyses of district health managers’ experiences with public feedback, views and concerns) with quality. We adopted the checklist in Supplementary Material 3, drawn from the study by Dixon-Woods et al. (2006) to assess the quality of the included studies. None of the 21 studies was excluded following the quality appraisal.

### Synthesis methodology

We adopted a framework approach to analysis (Walt and Gilson, 2014; Parashar *et al.*, 2020a) drawing on Gaventa’s power cube (Gaventa, 2005) and Norman Long’s concept of actor life-worlds (Long, 2003). Our power analysis was informed by the understanding that power is a dynamic resource that can be shared and used by individuals and groups (Veneklasen and Miller, 2002; Gaventa, 2005). Gaventa’s power cube was a good fit, given its relevance for researchers with applied interests and as we hoped through our power analyses to generate ideas about how responsiveness might be deepened. Norman Long’s actor-oriented perspective on power illustrates how the lived experiences of actors, their interactions and power struggle shape policy implementation (Long, 2003). The combination of these two power frameworks supported analysis that both (1) identified structural and organizational power (Gaventa’s power cube) and (2) considered power at the micro level to understand power differentials and struggles between actors (Long’s actor interface analysis) and how both impacted the practice of responsiveness. We focused on actors in a bid to be responsive to calls for more actor-centric HPSR (Sheikh *et al.*, 2014; Topp *et al.*, 2021).

Table 2 presents a summary of Gaventa’s power cube and illustrates the three dynamic and interacting dimensions of power: levels, spaces and forms of power (Gaventa, 2003, 2005). Spaces for power refer to mechanisms or channels where actors can influence decisions or policy. These spaces are shaped by power relations that determine who can participate in them (Gaventa, 2003, 2005). Levels of power include local, national and international arenas. The forms of power build on Lukes’ 1974 three dimensions of power (Lukes, 1974, 2004) and encompass visible, hidden and invisible forms of power.

**Table 2.** Gaventa’s dimensions of power

	Details
Spaces for power	
Closed spaces	Decisions are made by a set of actors behind closed doors. Within the state, this might be in the form of elites, bureaucrats or elected representatives making decisions without the involvement of the broader public
Invited spaces	Spaces are created into which people (as users, citizens or beneficiaries) are invited to participate by various kinds of authorities such as governments, NGOs
Claimed spaces	Spaces formed by less powerful actors from or against the power holders. These may form as a result of popular mobilization, or around identity or issue-based concerns, or like-minded people coming together to debate issues
Forms and visibility of power	
Visible	Definable and observable decision-making. Includes formal structures of authority, institutions and procedures of decision-making
Hidden	Certain powerful people and institutions maintain their influence by controlling who gets to the decision-making table and what gets on the agenda. Mainly operates by excluding certain people and devaluing the concerns of less powerful groups
Invisible	Shapes the psychological and ideological boundaries of participation. Significant problems and issues are not only kept from the decision-making table but also from the minds and consciousness of the different players involved, even those directly affected by the problem. May be perpetuated by socialization and cultural processes that define what is acceptable
Levels of power	
Global	Decision-making based on agreements and treaties by global and international bodies such as WHO and World Bank
National	Decision-making at the macro level, to include national governments and development partners
Local	Decision-making at the subnational level might include counties, districts and provinces down to the community level

Source: Gaventa, 2005; Gaventa and McGee, 2013.

Long’s actor interface analysis supported an in-depth exploration of power struggles between actors (Long, 2003; Parashar *et al.*, 2020a). According to Long, the points of interaction between actors in relation to a policy can be understood as actor interfaces. These interfaces are shaped by intersecting ‘actor life-worlds’, a term that refers to the lived experiences of actors. The formation of these life-worlds is dynamic and linked to the contexts of actors’ lives (Long, Long, 2003). Table 3 presents a summary of these contexts including their associated elements. The contexts include knowledge and power relationships in society and organizations, personal characteristics and world-views influenced by social–cultural–ideological standpoints.

**Table 3.** Actor life-worlds

	Broad dimensions of actor life-worlds		
	Power relationships	Personal concerns or characteristics	Social/cultural/ideological world-views
Elements	Social positions or status, authority, organizational/institutional hierarchy, technical/professional expertise, resourcefulness, gender, caste, class relations	Individual interests, motivation, identity, image, recognition, previous experiences, cognitive and behavioural traits, situations in personal lives, understanding	Values, norms, beliefs, moral standing, religious views, organizational/institutional norms and culture

Source: Long, 2003; Parashar *et al.*, 2020a.

Power practices ranging from domination, collaboration, negotiation and resistance to contestation may be observed within the actor interfaces (Long, 2003; Parashar *et al.*, 2020a). Table 4 elaborates more on these power practices. Concerning Gaventa’s power cube, we anticipated that these power practices may be observable across the forms and within the spaces and levels of power.

**Table 4.** Power practices

Power practice	Definition and illustration of where observed
Domination	Certain actors holding positional power (managerial and professional) over other actors
Negotiation	Occurs when actors are partially aligned with another actor’s decisions or actions
Collaboration	Actors work together to support an action or decision
Contestation	Opposition between two actors interacting at an interface
Resistance	Actors object to or oppose a decision or action of another actor

Source: Long, 1999; Parashar *et al.*, 2020a.

### Data extraction and derivation of themes

We first read and re-read the studies to identify raw data for the synthesis. A data extraction Excel sheet was devised to assist in systematically identifying characteristics of the papers, study objectives and actors described in the papers. The template for extraction of content from the review articles also included columns for the feedback channel, the content of feedback, processing of feedback, responses generated from feedback and composition of the DHMT (see Supplementary Material 4 for the full list of articles and sample of extracted content). This content was useful to answer the overarching review question. Texts were also uploaded onto Nvivo version 12, to support line-by-line coding of the primary texts. For the power synthesis, we drew on concepts from Gaventa’s power cube and Long’s interface analysis to code for data on actors with whom the DHMT interacted in receiving and responding to feedback, spaces and levels where feedback was received, discussed and responded to, forms of power observed within the DHMT or influencing the DHMT in receiving and responding to feedback, power practices by individuals or groups of actors, effects of power practices and actor life-worlds underpinning practices of power. Data for actor life-worlds were obtained by coding for actor life-world dimensions and then sub-coding for the characteristic elements of actor life-worlds described in Table 3. During coding, we considered data from all sections of an article, including author judgements (author’s insights into reported data) (Gilson, 2014). This included information on context reported by the authors that was useful for our understanding of findings on power.

The process of deriving themes combined deductive and inductive approaches. Themes were developed drawing on the conceptual and power framework, and all studies were coded according to which element of the frameworks they addressed. In addition, new topics were developed and incorporated as they emerged from the reviewed articles. To support comparison across papers, data extracted from various sections of the primary studies were entered into charts. NK developed a written summary to accompany the charts for discussion and agreement with the authorship team. Analysis of the evidence presented in the charts formed the basis for an overarching synthesis about how power might influence the functioning of a space within the health system where public feedback is received and responded to.

### Study scope

This synthesis was limited to papers that discussed receiving and/or responding to public feedback by the DHMT. It is constrained by the limits of the included papers. For example some of the revwied articles did not alwsy link public feedback to a response generated at DHMT level. The paper focuses on the practice of responsiveness by DHMTs and power dynamics between multiple actors influencing DHMT responsiveness; other factors such as the design of responsiveness policy and guidelines are not included here, although we recognize that these may influence DHMT handling of public feedback.

### Characteristics of the articles

**Figure 2.**
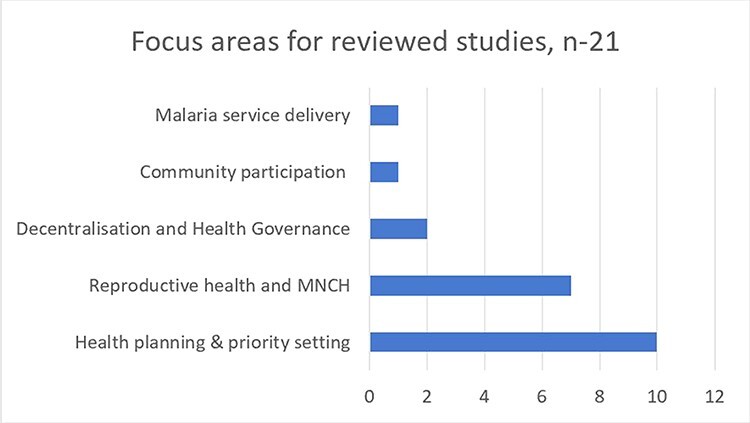
Focus areas reported on within reviewed articles

The 21 articles reported studies that mainly used qualitative data collection methods such as in-depth interviews, focus group discussions, observation and document review. The studies formed two broad categories: those that examined health system functioning with some consideration of public feedback at the district level (Kapiriri *et al.*, 2003; Tuba *et al.*, 2010; Maluka, 2011; O’Meara *et al.*, 2011; Cleary *et al.*, 2014; Van Belle and Mayhew, 2016; Nyikuri *et al.*, 2017; Tsofa *et al.*, 2017; McCollum *et al.*, 2018; Henriksson *et al.*, 2019; Razavi *et al.*, 2019; Jacobs and Baez Camargo, 2020; Mukinda *et al.*, 2020; Parashar *et al.*, 2020b) and intervention studies that reported on efforts to enhance inclusion of and response to public feedback in the priority setting (Maluka *et al.*, 2011; Byskov *et al.*, 2014; Zulu *et al.*, 2014), including through social accountability approaches (Blake *et al.*, 2016; George *et al.*, 2018; Boydell *et al.*, 2020; Butler *et al.*, 2020). The reviewed studies reported on experiences from a range of geographical contexts spanning sub-Saharan Africa (18 of 21 papers), India (2/21) and Central Asia (Tajikistan) (1/21) and addressed a range of issues from general health governance to specific service delivery areas (see Figure 2).

Regarding the governance contexts in which the DHMTs are operated, 15 out of 21 articles mentioned a decentralized context. However, in the majority of these 15 studies, there was inadequate detail to judge the form of decentralization, with only six studies, three in Kenya (Nyikuri *et al.*, 2017; Tsofa *et al.*, 2017; McCollum *et al.*, 2018) and three in Uganda (Kapiriri *et al.*, 2003; Razavi *et al.*, 2019; Boydell *et al.*, 2020), clearly stating and providing details of a devolved context.

## Results

The results of the literature review and synthesis are presented in two broad parts. The first part describes the processes of receiving, processing and responding to public feedback at the DHMT level, including specific feedback channels utilized by the public and the content of public feedback. The second part focusses on the exercise of power by the DHMTs themselves and actors with whom DHMTs interacted. Concerning how DHMTs managed public feedback, findings from the review suggest that a mix of formal and informal channels was utilized to receive public feedback, but there was little analysis (or processing) of feedback. Feedback channels in the reviewed studies appeared to exclude vulnerable groups, and in the few instances where responses were generated, there was little communication to the public. These elements of responsiveness are presented in more detail in subsequent sections.

### Processes of receiving, processing and responding to public feedback by DHMT

#### How DHMTs received feedback from the public?

From the studies, we identified five broad categories of channels through which DHMTs received feedback from the public (Box 1). Four of these categories were formal mechanisms established in country policy and guidelines. The last category, informal feedback channels, was more commonly reported in contexts where challenges were faced in the functioning of the formal mechanisms.

Box 1. Feedback channels through which DHMTs received public feedback

Formal feedback mechanisms

District-level participatory channels

1. District stakeholder forums (Kapiriri *et al.*, 2003; Van Belle and Mayhew, 2016; Razavi *et al.*, 2019)
2. District health councils (Tuba *et al.*, 2010)
3. Council health boards (Maluka *et al.*, 2011)

Ward- or village-level participatory channels

1. Neighbourhood committees(Zulu *et al.*, 2014)
2. Health unit management committees
3. Public health committees (Kapiriri *et al.*, 2003)
4. CORPs^2^ (O’Meara *et al.*, 2011)
5. VHTs (Boydell *et al.*, 2020), public participation meetings (Kapiriri *et al.*, 2003; McCollum *et al.*, 2018; Razavi *et al.*, 2019)
6. LAGs^3^ (Cleary *et al.*, 2014)
7. Community-based Health Planning Services (Van Belle and Mayhew, 2016)

Peripheral facility-level channels

1. Clinic committees and complaint management systems (Mukinda *et al.*, 2020)
2. Suggestion (Tuba *et al.*, 2010) and complaint boxes (Van Belle and Mayhew, 2016)

Social accountability interventions supported by NGOs

1. Community scorecards (George *et al.*, 2018)
2. Facility report cards (Blake *et al.*, 2016)
3. Community dialogue meetings (Butler *et al.*, 2020)
4. CBOs/village organizations (Jacobs and Baez Camargo, 2020)

Informal feedback mechanisms

1. Direct calls to DHMT members (Van Belle and Mayhew, 2016)
2. Phone calls to influential actors (Parashar *et al.*, 2020b) * *
3. Public airing of service delivery concerns on radio (Van Belle and Mayhew, 2016)

Despite policy provisions, several studies reported variations in the extent to which public feedback successfully reached DHMTs (Kapiriri *et al.*, 2003; Maluka, 2011; Nyikuri *et al.*, 2017; McCollum *et al.*, 2018; Razavi *et al.*, 2019). Poor attendance at budgeting and planning meetings by community members was cited as a challenge to including public feedback in the priority setting (Kapiriri *et al.*, 2003; Maluka, 2011; McCollum *et al.*, 2018; Razavi *et al.*, 2019). In Kenya, a lack of capacity and clarity about who was responsible for budgeting and planning within the department of health in the newly decentralized context constrained inclusion of public priorities (Nyikuri *et al.*, 2017). In Ghana, the absence of ‘functioning’ mechanisms within the district bureaucracy combined with a focus on vertical (to regional managers) and horizontal (to NGOs) accountability limited public accountability (Van Belle and Mayhew, 2016). Similarly, in South Africa, there was a predominance of internal bureaucratic accountability initiatives focused on the performance of health-care providers at the expense of accountability to the public (Mukinda *et al.*, 2020). Finally, in Tajikistan, NGO-supported community-based organizations (CBOs) at the village level had little leverage to demand feedback from the DHMT as they were directly linked to NGOs rather than the state mechanisms (Jacobs and Baez Camargo, 2020).

#### Who provided feedback and what was the content of the feedback?

The equity element of responsiveness requires consideration of which groups provide feedback and whether marginalized groups give feedback (WHO, 2000; Khan *et al.*, 2021). However, in the majority of papers reviewed, feedback was commonly reported as though voiced by a homogenous public. Several studies noted that vulnerable groups were often left out of priority-setting processes for the health sector (Kapiriri *et al.*, 2003; McCollum *et al.*, 2018; Razavi *et al.*, 2019), lacked representation in decision-making committees (Van Belle and Mayhew, 2016) or experienced barriers to voicing concerns about specific services such as reproductive health (RH) (Boydell *et al.*, 2020). These vulnerable groups were women, the youth, people with disability and adolescents (Kapiriri *et al.*, 2003; Van Belle and Mayhew, 2016; McCollum *et al.*, 2018; Boydell *et al.*, 2020). Four studies explored in some detail the factors that contributed to the exclusion of vulnerable groups in terms of priority setting (Kapiriri *et al.*, 2003; Van Belle and Mayhew, 2016; McCollum *et al.*, 2018; Boydell *et al.*, 2020). This is discussed in more detail in the section on power.

Of the 21 articles reviewed, only six included details about the content of public feedback (see Supplementary Material 5). Drawing on these papers, we identified four broad categories of public feedback: i) provider–client interactions, ii) infrastructure, staffing and commodity-related issues, iii) requests for the introduction of new services and iv) broader environmental and health system issues impacting health service uptakes.

#### Processing of public feedback

About a third (7/21) of the studies reported some form of analysis or consolidation of feedback at the district level (Maluka *et al.*, 2011; O’Meara *et al.*, 2011; Byskov *et al.*, 2014; Zulu *et al.*, 2014; Blake *et al.*, 2016; George *et al.*, 2018; Butler *et al.*, 2020). The details of processing public feedback in the reviewed studies are summarized in Table 5, which highlights that in the cluster of health sector priority-setting studies (Maluka, 2011; Maluka *et al.*, 2011; O’Meara *et al.*, 2011; Byskov *et al.*, 2014; Zulu *et al.*, 2014), we identified consolidation of community input at the facility level and then upward submission to the district level. Table 5 also highlights that practice differed from recommendations about processing arrangements. For example, review by a multi-stakeholder board was uncommon in Tanzania (Maluka, 2011), but public appeal of disseminated priorities hardly occurred across several countries (Maluka *et al.*, 2011; Byskov *et al.*, 2014; Zulu *et al.*, 2014).

**Table 5.** Processing of public feedback at the district level

Studies	Details of proposed processing of feedback received from the public	How processing played out in practice as reported in reviewed articles
Priority-setting studies Maluka, 2011; Maluka *et al.*, 2011; O’Meara *et al.*, 2011; Byskov *et al.*, 2014; Zulu *et al.*, 2014	Consolidation of community priorities shared from the community level, upwards to facility and district levels	Community priorities were consolidated and shared upwards to the PHC facility level and then to the district level
Maluka, 2011	‘Review by a multi-stakeholder board’ comprising community representatives to check for inclusion of community priorities	This board was often bypassed because they did not meet frequently. (1) The board also lacked the capacity to scrutinize budgets and plans for the inclusion of community priorities
O’Meara *et al.*, 2011	‘Community priorities were considered in relation to district targets’ (which were shared in a top-down process informed by national indicators)	The community priorities were excluded if they did not align with the national indicators and district targets. District targets were developed in a separate process that was linked to national indicators
Maluka *et al.*, 2011; Byskov *et al.*, 2014; Zulu *et al.*, 2014	‘Information provision to the public to give room for appeal’ before formally adopting the district plans	The public did not appeal any of the proposed priorities shared
Social accountability studies Blake *et al.*, 2016; George *et al.*, 2018	Quantitative analyses of facility and community scorecards results	
Blake *et al.*, 2016; Butler *et al.*, 2020	Combination of quantitative and qualitative summaries of findings from multiple feedback mechanisms	

Finally, Table 5 shows the processing of public feedback in the cluster of studies reporting on social accountability interventions. This processing was supported by NGOs and mainly entailed ‘quantitative analyses’ of facility scorecard results (Blake *et al.*, 2016) and village-level report cards (George *et al.*, 2018) to develop summaries of data collected from service users. In two studies, conducted in Malawi (Butler *et al.*, 2020) and Uganda (Boydell *et al.*, 2020), feedback from multiple mechanisms was integrated, combining both qualitative and quantitative analyses. Across all four studies describing social accountability interventions, public feedback was shared with district health managers (Blake *et al.*, 2016; George *et al.*, 2018; Boydell *et al.*, 2020; Butler *et al.*, 2020), who responded as described later. Notably, processing of feedback was not done within the DHMTs in these studies. Instead, NGOs performed the analysis (Table 5) and shared the findings with the DHMT.

#### Responses to public feedback

Seven studies discussed some detail on responses to public feedback. One study conducted in Zambia highlighted district managers’ ‘selection’ of issues to respond to, based on their perception of what they could influence. For example, there were instances when DHMTs simply ‘took no action’ despite receiving public feedback. This was reported in the study by Tuba et al. (2010) regarding complaints related to waiting times and health provider behaviour such as rudeness to the public (Tuba *et al.*, 2010). However, the same district managers responded to complaints about overpriced nets at the facility level by collaborating with an NGO to set up a monitoring system for tracking the sale of insecticide-treated nets (Tuba *et al.*, 2010). In Ghana, the DHMT also ‘took no action’ in response to public feedback despite the public’s efforts to express their service delivery concerns through radio and calls to DHMT members (Van Belle and Mayhew, 2016). Across both studies, there was a failure to acknowledge complaints from the public, and thus, no responses were generated at all. In the study by O’Meara *et al*., response to public feedback was in the form of community priorities being adopted only if they aligned with national targets (O’Meara *et al.*, 2011). All other priority-setting studies (Maluka *et al.*, 2010; 2011; Byskov *et al.*, 2014; Zulu *et al.*, 2014) simply did not discuss whether community priorities eventually informed district plans.

Four other studies highlighted specific responses generated at facility, community or district levels. In these studies, the reported responses appeared to have had system-level effects. They included provision of a vehicle to improve the referral system within the district (Blake *et al.*, 2016), increasing budget allocations for family planning (FP) and RH services (Boydell *et al.*, 2020), inclusion of identified service needs in the financial plan for the subsequent year (George *et al.*, 2018) and improvements in facility infrastructure and initiation of service delivery in defunct facilities (George *et al.*, 2018). These four studies also reported escalating some feedback to the regional and national level, but responses from these higher levels were not discussed.

### Manifestations of power in processes of receiving and responding to public feedback at the district level

In this section, our findings related to the exercise of power are presented in three subsections. First, we consider where actor interfaces (points of interactions between actors) are situated and how power was exercised within and across Gaventa’s levels and spaces of power. Second, we explore the forms of power observed at the actor interfaces, including their linked power practices, and the actor life-worlds underpinning the identified forms and practices of power. This approach allowed further deconstruction of the exercise of power to reveal the agency and motivations of actors. Third, we present findings on the effects of the observed power dynamics on DHMT handling of public feedback. All these findings are summarized in Table 6, which presents synthesis findings about the processes of receiving and responding to public feedback. For the various instances drawn from the reviewed articles in Table 6, we highlight the observed form of power, the level and the space where this power was observed to be exercised. Table 6 also presents the associated practices of power, underpinning actor life-worlds and the effects on responsiveness for each of the instances highlighted.

**Table 6.** Actor life-worlds underpinning power practices and the effects of power practices

Incident	Form of power	Levels and space of power	Actor interface	Practice of power	Underlying actor life-world			Effects of power practices
Receiving public feedback					Power relationships	Personal life concerns or characteristics	Social/cultural/ideological world-views	
DHMT’s use of HFCs to collect community priorities for district planning. National guidelines had recommended use of CORPs who would have had wider reach than the pre-existing HFCs but had not trained the CORPs (O’Meara *et al.*, 2011)	Visible power	District level: DHMT and HFC (invited space)	DHMT/HFC members	‘Collaboration’ between community representatives and DHMTs to identify community and facility priorities for inclusion in district plans	DHMT status within the district health system as decision-makers; DHMT resourcefulness in using existing community structures when those proposed in national guidelines were unavailable			Promoted responsiveness to local priorities by supporting implementation of community participation in priority setting despite inadequate resources
Control over priority-setting information available to other DHMT members and stakeholders (by those within the core DHMT) (Maluka, 2011)	Visible power	District level Within the DHMT space	DHMT core team members/other DHMT members and stakeholders	‘Domination’ of priority-setting process by District Medical Officer (DMO)	Access to schedule and information on priority setting			Undermined responsiveness to public needs: closed the space for collection and discussion of stakeholder (including the public) input into priorities
Failure to engage existing community structures to learn about community views and priorities; failure to acknowledge public concerns shared via direct calls to DHMT members and via radio (Van Belle and Mayhew, 2016)	Visible power	District level DHMT and CHPS (invited space)	DHMT/public	‘Domination’ over the public by failing to acknowledge their concerns and failing to use existing community structures to learn about public concern	DHMT status and authority within the district health system as decision-makers		Little value attributed to feedback from the public/community (AJ)	Undermined responsiveness-locked out public feedback
Control over resources such as funding, vehicles and ambulances by core DHMT members limiting availability of these resources to other DHMT members who were required to conduct peripheral facility visits for support supervision (Jacobs and Baez Camargo, 2020)	Visible power	District level; within DHMT space	DHMT core team members/other DHMT members	‘Domination’ of funds collected from district hospital by district hospital director and his core team	District hospital director’s position and access and control over funds generated at the hospital level			Undermined responsiveness—DHMT members could not conduct visits to peripheral health facilities to monitor service delivery and learn about public feedback
	Visible power	District level; within DHMT space	DHMT core team members/other DHMT members	‘Contestation’ over control of ambulances between district hospital director and DHMT members	District hospital director’s position and access and control over funds generated at hospital level	Personal situations of DHMT members who earned low wages and had little resources for work		The interaction of a resource constrained DHMT, coupled with personal experiences of below subsistence wages oriented DHMTs away from attention to public feedback and focused their attention on rent-seeking, which in turn undermined public trust in the district officials
Selective mobilization of the public by politicians for participation in the priority setting (Kapiriri *et al.*, 2003)	Hidden	Community (ward and village) Public participation forum (invited space)	Politicians/public	‘Domination’ of the public by politicians	Status within the governance structure and access to information about priority setting	Personal interest to advance individual political careers		Undermined inclusivity in the priority setting process—Members of the public failed to attend public participation forum due to a perception of ‘being exploited’
Failure to educate the public on their rights and responsibilities in priority setting (McCollum *et al.*, 2018)	Hidden	Community level (ward) Public participation forum (invited space)	Politicians/public	‘Domination’ of the public by politicians			Belief among politicians that by sharing power (by empowering the public with information) they would themselves lose power	Members of the public were reluctant to attend public participation forums ‘undermining functioning’ of the forum as a ‘feedback mechanism’
Low participation of public in appealing priorities agreed upon at the district level (Maluka *et al.*, 2011; Zulu *et al.*, 2014)	Invisible power	District level	DHMT/public				Culture among the public of not questioning authority (Zulu *et al.*, 2014). Belief among members of the public that health providers were best placed to make priority-setting decisions (Maluka *et al.*, 2011)	Undermined inclusivity of priority-setting process
Low attendance of public participation meetings, for priority setting (McCollum *et al.*, 2018; Kapiriri *et al.*, 2003)	Invisible power	Community (ward and village) level	Politicians/public	‘Resistance’ by the public to participate in community governance or feedback mechanisms involving politicians		Previous experiences of neglect of vulnerable groups especially youth, women and communities from marginalized geographical areas	Patriarchal norms and culture kept women from attending as meetings were held during the day when they had household chores (McCollum *et al.*, 2018; Kapiriri *et al.*, 2003) Women also failed to attend due to concerns about lacking ‘proper’ clothes (Kapiriri *et al.*, 2003) Belief that historical neglect by government would continue to persist despite governance changes (McCollum *et al.*, 2018) Belief that politicians were paid to identify local priorities and so ought to do priority setting by themselves (Kapiriri *et al.*, 2003)	Sustained continued exclusion of vulnerable groups from priority-setting processes
Rejection of engagement with local politicians as a means of providing feedback on FP and RH services (Boydell *et al.*, 2020)		Community (village) and facility level	Politicians/public	‘Resistance’ by the public to sharing feedback on FP and RH services with political representatives		Previous experiences of last-minute participation by political actors to gain political mileage		Contributed to the use of feedback mechanisms that members of the public perceived not to be exploitative, therefore enhancing responsiveness
The CHSB, comprising community representatives and other stakeholders, was frequently bypassed when district plans were developed by the CHMT (DHMT equivalent) (Maluka, 2011)	Visible power		DHMT/public	‘Domination’ of district health managers over community representatives	Access to information; professional expertise on the priority setting			Undermined responsiveness by locking out inclusion of public input into district plans
CHMT members presented district plans to the Full Council late in the priority-setting process, leading to little scrutiny by CHSB members to ensure inclusion of community priorities; Full Council members also did not have adequate capacity (lacked training) to examine the documents (Maluka, 2011)	Visible power	District level; spaces DHMT and CHSB (invited space)	DHMT/public	‘Domination’ of district health managers over community representatives	Professional expertise of CHMT members in contrast to Full council members who did not have sufficient knowledge about priority setting			Undermined responsiveness by locking out inclusion of public input into district plans
Responding to public feedback								
DHMT members were present and showed support for NGOs adaptation of local community mechanisms for the public to raise concerns related to maternal health service delivery; DHMT members also took up issues for action at the district level and escalation to the national level (Butler *et al.*, 2020)	Visible power	Interface spanning village and district levels Community dialogue forum (invited space)	DHMT/NGO	‘Collaboration’ between NGOs and DHMTs to support public sharing of feedback by community members by attending dialogue meetings (DHMT) and providing funding for these meetings (NGOs)	Access to resources (funding) by NGO to support functioning of feedback mechanisms Status of DHMT members to take action on certain forms of feedback and to escalate others for action at the national level		Pre-existing values of openness and transparency (AJ)	Collaboration led to the identification and resolution of multiple issues at community and district levels, contributing to ‘services more aligned to public needs’ and also to an increase in the feedback mechanisms as members of the public became more confident about voicing their concerns
DHMT members failed to act on feedback shared with them about HCW provider behaviour; long waiting times and prioritizing politicians and their families to receive services first (Tuba *et al.*, 2010)	Invisible power	District level	DHMT/public	‘Domination’ over members of the public by failing to act on concerns raised	Authority of the DHMT undermined by organizational hierarchy, which limited decision space to sanction frontline health providers	Previous experience of politicians instituting forced transfers	Belief among DHMT members that different ‘categories’ of people (e.g. politicians) should be treated differently Belief that community members were not ‘legitimate’ stakeholders due to lack of professional training	The public was unable to elicit responses from the DHMT for feedback shared
Members of the public made calls to politicians to access expected benefits of a Mother and Child Safety scheme	Visible power	Hospital level	Beneficiaries/frontline service providers and health facility managers	‘Contestation’—Utilization of personal connections with influential people to share complaints/concerns about service delivery	Resourcefulness among members of the public who leveraged relationships with individuals with higher social status to access their entitlements		Belief among members of the public in patients’ rights and entitlements (AJ)	Beneficiaries’ actions of negotiations tilted the power dynamic in favour of the patients, resulting in action in response to patients’ demand for better services
In-kind gifts to frontline providers given by members of the public to generate a form of answerability at the local facility level (Jacobs and Baez Camargo, 2020)	Visible power	Peripheral facility level	Public/frontline service provider	‘Negotiation’—Members of the public recognized the lack of resources in health facilities and provided support by offering repair services for clinic building and gave produce from farms as gratitude payments	Resourcefulness among members of the public		Lack of belief in possibility of answerability from district-level managers and state actors (AJ)	Created an informal tool of enforceability and a sense of answerability between frontline providers at the peripheral facilities and the public, which excluded DHMT
Exclusion from district plans of community priorities that did not match national targets by national health managers (O’Meara *et al.*, 2011)	Invisible power	District level	DHMT and public/national health managers	‘Domination’ of national health managers over district health managers			‘Institutional norm’ of the top-down priority setting	Despite DHMT efforts to engage HFC members in the priority setting, emphasis on national-level indicators left many local priorities unaddressed in the final district plans
Culture of adopting MoH priorities due to the historic centralized priority setting prior in decentralised contexts (Kapiriri *et al.*, 2003; Maluka, 2011; Maluka *et al.*, 2011; Byskov *et al.*, 2014)	Invisible power	District level	DHMT and public/national health managers	‘Domination’ of national health managers over district health managers			‘Institutional norm’ of top-down priority setting	Undermined generation of responses to locally identified needs
Bureaucratic practice within health systems: national and regional health managers often modified district plans after they had been submitted to them leading to exclusion of priorities raised by the public (Zulu *et al.*, 2014)	Invisible power	District level	DHMT/regional and national health managers	‘Domination’ of national health managers over district health managers			‘Institutional norm’ of top-down priority setting; ‘Belief’ among health managers that the public did not have the necessary skills to participate in priority setting	
Multiple internal performance accountability mechanisms, with few public accountability mechanisms and more attention paid to the internal performance mechanisms (Mukinda *et al.*, 2020)	Invisible power	District level	Public and health providers and district managers				‘Organizational culture’ of bureaucratic compliance with top-down performance indicators	Orienting DHMT attention away from public feedback mechanisms

AJ = author judgement; CHMT = Council Health Management Team; CHSB = Council Health Services Board; CHPS = Community-based Health Planning Services; MoH = Ministry of Health

#### Multiple actors and a wide range of interactions across health system levels and spaces in relation to receiving and responding to public feedback

Table 6 highlights the multiple interactions between DHMTs and various actors in the processes of receiving and responding to public feedback. These actors included: community representatives, individual community members, political actors, regional and national health managers and NGOs. At these points of interaction, we identified actor interfaces situated both within and across Gaventa’s levels and spaces of power. Importantly, despite having a formal mandate to oversee health service delivery and planning, many DHMTs, even in decentralized countries, had limited decision-making autonomy. At the interface between DHMTs and regional/national health managers, the higher-level managers often ‘dominated’ the planning process. For example, DHMTs could not make final decisions on plans and budgets at the district level as they were required to follow national-level guidelines, with little room for local priorities. Changes to district plans were also often made at the regional or national level (Maluka, 2011; O’Meara *et al.*, 2011; McCollum *et al.*, 2018; Henriksson *et al.*, 2019). DHMTs also operated in contexts of resource scarcity illustrated by unpredictable and inadequate disbursements of funds from national or regional levels (Van Belle and Mayhew, 2016; Nyikuri *et al.*, 2017; Jacobs and Baez Camargo, 2020), understaffing and low supplies of commodities for the primary health-care (PHC) facilities they supervised (Tuba *et al.*, 2010; Jacobs and Baez Camargo, 2020). NGOs operating at the district level sometimes filled a few of these resource gaps (Tuba *et al.*, 2010; Van Belle and Mayhew, 2016; Jacobs and Baez Camargo, 2020), forming an interface with the DHMT. However, there were drawbacks related to NGO ‘collaboration’ with DHMTs. For example, in Ghana, Van Belle and Mayhew reported that the DHMT in the study districts engaged with three NGOs frequently, leaving little opportunity for engagement and inclusion of the public in planning activities (Van Belle and Mayhew, 2016).

In several studies, we identified clusters of DHMT members working closely together (Maluka, 2011; Zulu *et al.*, 2014; Van Belle and Mayhew, 2016; Jacobs and Baez Camargo, 2020), which we judged to be ‘closed spaces’. Here, decision-making occurred with little or no consultation with other DHMT members and stakeholders. These ‘core teams’ comprised individuals with leadership roles in the DHMT or with resource allocation–related roles. In two priority-setting studies, these core teams dominated health planning by withholding access to district plans such that there was inadequate time for other DHMT members’ or stakeholders’ views to be incorporated into the plans before upward submission to the national level (Maluka, 2011; Zulu *et al.*, 2014). In one of the broader governance studies in Tajikistan, a core team^4^ within the DHMT concentrated resources at the district hospital and denied other DHMT members’ resources for their activities, including for visiting peripheral facilities where they could have picked up issues related to public feedback (Jacobs and Baez Camargo, 2020).

At many of the interfaces shown in Table 6, the public was often the less powerful actor. However, they were not passive actors; when the ‘invited spaces’ failed to provide an avenue for public feedback to reach the DHMT, the public attempted to evolve ‘claimed spaces’ where they voiced complaints. For example, in Ghana, where DHMTs were more focused on reporting upwards to their regional managers and horizontally to NGOs, the public used radio and increased litigation to share complaints and concerns about the health system (Van Belle and Mayhew, 2016). In Tajikistan where autocratic rule had undermined formal voice mechanisms at the interface between health providers and the public, the public provided in-kind contributions at under-resourced peripheral facilities, creating a degree of answerability for service provision between the community and frontline providers (Jacobs and Baez Camargo, 2020). These claimed spaces seemed to have mixed results in tilting power towards the public. In the Ghanaian study, the authors reported that despite the public’s efforts to share feedback in new ways, such as radio, and through direct calls to DHMT members, there was a failure to acknowledge this public feedback by the DHMTs who failed to respond (Van Belle and Mayhew, 2016), while in the Tajikistan study, the authors observed that it was possible that the public may have been coerced by frontline health-care workers (HCWs) into providing in-kind contributions that reportedly contributed to higher facility-level responsiveness to the concerns of the public (Jacobs and Baez Camargo, 2020). In the study by Parashar *et al*., the public was more successful with their claimed space, as they leveraged connections to powerful and influential actors (‘power relationships’) to access their entitlements as beneficiaries in a mother–child safety programme (Parashar *et al.*, 2020b).

#### Forms of power and their linked power practices were underpinned by varying and interacting actor life-worlds in relation to receiving and responding to public feedback

In this section, we explore the various forms of power and specific practices of power identified from the reviewed studies. Furthermore, we also present what actor life-world supported the identified exercise of power. These instances of exercise of power are highlighted in Table 6, which shows that visible power was a dominant form of power associated with both positive and negative power practices. For example, the DHMT, given its formal mandate and managerial authority over health planning and service delivery, was a space where ‘visible power’ was commonly exercised. Some of the power practices linked to visible power include, for example, in the intervention studies implementing social accountability initiatives, ‘collaboration’ between NGOs and DHMTs, as both exercised visible power to identify and respond to public feedback. NGOs drew on their resources and technical expertise to support the functioning of community scorecards (Blake *et al.*, 2016), facility report cards (George *et al.*, 2018), village health teams (VHTs) and local civil society organizations (CSOs) (Boydell *et al.*, 2020; Butler *et al.*, 2020) to collect public feedback, while DHMTs exercised their positional power to respond to some of the issues raised by the public (see the section on ‘Responses to public feedback’). However, there were also instances where DHMT members used their power in ways that undermined responsiveness to public feedback. For example, in Ghana, DHMTs ‘dominated’ the public and community representatives by failing to acknowledge public feedback despite the public’s efforts to use new channels like radio, litigation and direct calls to the DHMTs to share their concerns (Van Belle and Mayhew, 2016).

In several studies, ‘visible power’ flowed in a top-down manner and the DHMT was commonly ‘dominated’ by national (Maluka *et al.*, 2011; O’Meara *et al.*, 2011; Zulu *et al.*, 2014), regional (Maluka *et al.*, 2011) and political actors (Nyikuri *et al.*, 2017; Tsofa *et al.*, 2017; McCollum *et al.*, 2018; Razavi *et al.*, 2019). At the national/DHMT, regional/DHMT and politicians/DHMT interfaces, we noted domination underpinned by ‘relationships of power’ rooted in organizational hierarchy (Maluka, 2011; O’Meara *et al.*, 2011; Byskov *et al.*, 2014; Zulu *et al.*, 2014) and control over resources (Maluka, 2011; Nyikuri *et al.*, 2017; Tsofa *et al.*, 2017; Razavi *et al.*, 2019). For example, in Kenya, there was reportedly little inclusion of sub-county health managers and the public in the health priority setting despite recent decentralization (Nyikuri *et al.*, 2017; Tsofa *et al.*, 2017; McCollum *et al.*, 2018). Decentralization had created semi-autonomous counties headed by political leaders. At the interface between health managers and politicians, both county and sub-county health managers had little room to challenge decisions made by politicians or their appointees (McCollum *et al.*, 2018). SCHMTs (DHMT equivalent) also experienced significant resource constraints, which made it difficult for them to learn about public feedback at the facility level (as they could not conduct timely support supervision visits) or in stakeholder meetings (Nyikuri *et al.*, 2017; Tsofa *et al.*, 2017; McCollum *et al.*, 2018). In this case, SCHMTs (and the department of health) were ‘dominated’ by higher-level county actors who concentrated resources at the county level.

We also identified ‘hidden power’ sometimes influencing whose (and what) feedback DHMTs received. In the priority-setting studies, powerful actors controlled public participation processes. In Uganda, at the public/politicians interface, politicians exercised hidden power by selectively mobilizing rich community members, while the youth and poorer community members were invited only after decisions regarding project costs and plans had been made (Kapiriri *et al.*, 2003). In Kenya, despite having the mandate to mobilize all community members for public participation, politicians made little effort to educate the public on their rights to participate in the priority setting and how to do so, perpetuating low public awareness and participation in the priority setting (McCollum *et al.*, 2018). In these exercises of hidden power, politicians commonly ‘dominated’ the public, a power practice underpinned by two interacting life-worlds. One was ‘power relationships’ rooted in politicians’ positions of authority and access to information. Second was the ‘personal concerns’ of politicians who wanted to appeal to their voter base and retain political power. In a Kenyan study, politicians reportedly prioritized resource allocation to areas where they had political support to secure votes or repay political promises (McCollum *et al.*, 2018). Similarly, in a social accountability intervention study reporting findings from Uganda, local politicians were perceived to sweep in to claim credit for changes arising from public feedback to garner political recognition (Boydell *et al.*, 2020).

In two priority-setting studies, the public reacted to domination by politicians ‘with resistance and contestation’. In Kenya and Uganda, the public perceived that their participation was tokenistic and resisted attendance of public participation meetings scheduled by local politicians (Kapiriri *et al.*, 2003; McCollum *et al.*, 2018). We judged this resistance to be underpinned by life-worlds shaped by ‘ideological world-views and personal characteristics’. For example, in Kenya, one of the marginalized communities held the belief (world-view) that public participation would not change the community’s circumstances given the ‘historic neglect’ of their region^5^ (McCollum *et al.*, 2018). In Uganda, beliefs of exploitation by politicians were linked to a view that politicians were paid to conduct public participation meetings, but they (politicians) then failed to pay the attendees. This contributed to low attendance of the public participation meetings by youth, the majority of whom was unemployed and felt that there should be tangible benefits from public participation (Kapiriri *et al.*, 2003). In addition, there were reportedly greater efforts by local politicians towards public mobilization during election periods, compared with the poor mobilization done for health sector planning. This resulted in feelings (personal concerns) among the public of ‘being forgotten’ by politicians after elections, which in turn underpinned their resistance to participation in public meetings for the priority setting.

‘Invisible power’ also appeared to influence both receiving and responding to public feedback. Constraints to receiving feedback that demonstrated invisible power included structural issues such as people’s illiteracy, lack of interest and awareness about the possibilities of participation (Maluka *et al.*, 2011; Zulu *et al.*, 2014; McCollum *et al.*, 2018), poverty and unemployment (Kapiriri *et al.*, 2003) and a culture of not questioning those in authority (Maluka *et al.*, 2011). In three studies, we also identified the influence of patriarchal norms in keeping women and youth from providing feedback on priority-setting (Kapiriri *et al.*, 2003; McCollum *et al.*, 2018) and RH services (Boydell *et al.*, 2020). In Uganda, men within the community perceived that women’s and youth’s participation in decision-making processes was ‘rebellious’ even though policy guidelines specifically identified women and youth as vulnerable groups whose views were to be included in all policy processes (Kapiriri *et al.*, 2003). Women failed to attend local council meetings because they could not afford to dress ‘appropriately’ and look ‘presentable’ at these meetings (Kapiriri *et al.*, 2003). In Kenya, women were often busy with household chores when public participation meetings were planned, and even when they attended, lacked the confidence to speak (McCollum *et al.*, 2018). In the Ugandan study by Boydell *et al*., women and youth agency in accessing and providing feedback about FP services was compromised by patriarchal and moral views that opposed FP use (Boydell *et al.*, 2020). In a different context, in Tajikistan, a history of autocratic leadership (characterized by absent electoral processes and local-level formal voice mechanisms) contributed to low expectations of answerability from local officials and district health managers (Jacobs and Baez Camargo, 2020). As a result, there was no attempt by the public to provide feedback to the DHMTs at all.

Concerning responding to public feedback, we identified the invisible power of bureaucratic hierarchy illustrated first, by a culture within DHMTs of adopting top-down priorities (Kapiriri *et al.*, 2003; Maluka *et al.*, 2011; O’Meara *et al.*, 2011; Byskov *et al.*, 2014; Zulu *et al.*, 2014). This culture persisted despite decentralization to the district in all of the priority-setting studies’ contexts. Kapiriri *et al.* (2003) described this as a tendency to plan ‘for’ the community rather than ‘with’ the community retained from the previously centralized health system (Kapiriri *et al.*, 2003). Second, two studies highlighted the focus of health managers and providers on internal performance requirements and horizontal accountability relationships (with NGOs) at the expense of responses to public feedback (Van Belle and Mayhew, 2016; Mukinda *et al.*, 2020). In South Africa, Mukinda *et al.* (2020) identified 19 formal and informal accountability mechanisms targeting district-level health managers and providers, the majority of which was related to performance accountability^6^ (Mukinda *et al.*, 2020).

From examining life-worlds (Table 6), it appears that ideological world-views mirror the exercise of invisible power by shaping actors’ views of what is acceptable. In several studies, the power practices DHMTs demonstrated in receiving and responding to public feedback were underpinned by beliefs and values, an element of ideological world-view. For instance, in Zambia, DHMT members failed to respond to community concerns related to discrimination in waiting times, due to a belief that *‘*it was fair that waiting times differed for different types of people’ (p 6) (Tuba *et al.*, 2010). One manager noted:

*Well in society, we have different people. Like even politicians can’t go in the queue. So that’s how you find, when people see that, they will start complaining. But it’s because maybe of one’s status in society, for example, the xxx (referring to a political position in the district) and other political leaders* (p 6) (Tuba *et al.*, 2010).

In the same study, decision-makers did not recognize the public as legitimate stakeholders because they lacked technical training (Tuba *et al.*, 2010). This together with the managers’ views previously suggests little value for public feedback. In contrast, in another Zambian study (Zulu *et al.*, 2014), DHMT members held values of openness and transparency that promoted collaboration between the DHMT and action research team to improve inclusivity in the priority setting. The DHMT drawing on their motto of ‘provision of health services in partnership with the community’ was able to quickly revive feedback channels such as HFCs, which had not been functioning before the intervention to enhance the inclusive priority setting (Byskov *et al.*, 2014; Zulu *et al.*, 2014). This DHMT’s world-view might have also been influenced by the implementation of a new decentralization policy, just before the reported intervention, which sought to increase the inclusion of varied stakeholder input, including that of the public (Byskov *et al.*, 2014; Zulu *et al.*, 2014).

Other actor life-world categories did not reflect a particular form of power as distinctly as ideological world-views. Nonetheless, they were useful to understand ‘reactions’ to the exercise of power by less powerful actors. For example, in several studies, we noted that the ‘personal concerns’ of DHMTs oriented them away from attention to public feedback. As reported by Tuba *et al*., the DHMT failed to act on complaints related to discrimination in waiting times and the provision of malaria supplies to politicians’ relatives (Tuba *et al.*, 2010). We judged this to be a power practice underpinned by the personal concerns of district health managers who feared that acting on these complaints would trigger workstation transfers instigated by politicians (Tuba *et al.*, 2010). In the Tajikistan study, DHMT members were paid such low subsistence wages that most of their visits to facilities were focused on rent-seeking and punitive actions against frontline providers for ‘wrong-doing’ (Jacobs and Baez Camargo, 2020). This domination over frontline providers, coupled with contestation over resources between the DHMT and district hospital director and his team actions, was shaped by an interaction of ‘power relationships and personal concerns’ (reflected by low wages) and had an overall effect of undermining public trust in district officials.

#### Effects of power practices and forms of power on receiving and responding to public feedback

Drawing on the experiences highlighted in Table 6, responsiveness appeared to be enhanced by collaborative power practices while contestation, domination and resistance often undermined receiving and responding to public feedback. For example, in Tanzania and Zambia, collaborative power practices drawing on the technical expertise of the action research team and the positional power of the DHMT led to the opening up of the closed space within the DHMT. The authors reported greater inclusion of other DHMT members and stakeholders in the priority setting, creation of an opportunity for the public to appeal and revitalization of community participation structures to support the collection of public feedback (Maluka *et al.*, 2011; Zulu *et al.*, 2014; Blake *et al.*, 2016).

In the social accountability intervention studies, NGOs drew on their resources, expertise and reputational power to support the functioning of feedback channels (Tuba *et al.*, 2010; Blake *et al.*, 2016; Boydell *et al.*, 2020; George *et al.*, 2018; Butler *et al.*, 2020), while DHMTs drew on their positional power to address some public feedback. These collaborative power practices created a virtuous cycle that enhanced responsiveness. First, the generation of visible responses to public views, such as increased access to commodities (Tuba *et al.*, 2010; Blake *et al.*, 2016; Boydell *et al.*, 2020), re-starting of services and infrastructural improvements in service delivery and addressing of kick-back practices at community and facility level (George *et al.*, 2018; Butler *et al.*, 2020), suggests that services were better aligned to community needs. Second, even where there was no immediate change in service delivery, responses such as DHMTs escalating issues to higher system levels or simply acknowledging community concerns increased the confidence of the public in voicing their needs. For example, in Malawi, a by-product of well-performing community dialogue forums in an NGO-supported district was that community members set up forums in other districts without NGO support (Butler *et al.*, 2020). Third, we identified reports of improved relationships between health providers and the public. For example, Blake *et al*. reported that an improved understanding of health providers’ difficult working environment contributed to the creation of a midwife award system by a local traditional leader (Blake *et al.*, 2016). In this study, one villager observed:

*Now I understand why they refer people. It’s because they are not at the level where they can take care of certain problems. Previously I thought they were not ready to help us* (p 376) (Blake *et al.*, 2016).

In contrast, domination, contestation and resistance, at the politicians/public interface at the district level, created a vicious cycle of low attendance that undermined the functioning of the public participation forum as a feedback channel and continued the marginalization of vulnerable groups (Kapiriri *et al.*, 2003; McCollum *et al.*, 2018). However, in the Ugandan study by Boydell *et al*., where the public also resisted working with politicians, domination by politicians was tempered by the presence of other feedback mechanisms^7^. In this study, the effects of contestation and resistance at the public/politician interface may also have been reduced by the district-level collaboration with an NGO that had adequate resources and the technical expertise to support VHTs (the feedback mechanism preferred by the public), which linked back to district health managers (Boydell *et al.*, 2020). Finally, in the Tajikistan study (Jacobs and Baez Camargo, 2020), the public, aware of the power struggles at the district level, evolved an informal answerability mechanism directly with frontline providers and shared feedback directly with NGO service providers. Both processes did not link back to the DHMT, and thus, they were locked out of the process of receiving public feedback.

## Discussion

This synthesis contributes to the literature on health system responsiveness by illuminating some of the actions taken by DHMTs in receiving and responding to feedback. However, the experiences reported in this synthesis have also highlighted weaknesses in the practice of responsiveness. These weaknesses included constraints to receiving feedback from the public (particularly vulnerable populations), little analysis (processing) at the DHMT level, inconsistent generation of responses and little communication to the public on generated responses. Few of the reviewed studies examined the role of power dynamics in DHMT responsiveness to public feedback in detail. Hence, we conducted a power analysis to understand our observations and generate ideas about how responsiveness might be strengthened. This synthesis adds to the emerging literature on responsiveness as a complex concept (Lodenstein *et al.*, 2017; Mirzoev and Kane, 2017; Khan *et al.*, 2021) pointing out the importance of actor interactions, power dynamics and varied elements of context as features of that complexity.

In the studies reviewed, DHMT members commonly exercised visible power linked to their managerial role. However, DHMT members were not uniformly empowered; some studies showed that core teams within the DHMT (closed spaces) had significant power linked to their access to resources and positions. The decisions and actions of these core teams influenced how the DHMT as a whole handled public feedback. We also noted that the public sector bureaucracy within which the DHMT operated held a form of invisible power embedded in its organizational culture that influenced to what extent DHMTs were willing and able to respond to public feedback. Several studies showed that politicians exercised hidden power, which influenced who was invited to share public feedback and what issues were included as priorities for discussion. Finally, we systematically identified invisible power as manifested in the subconscious influence of social norms, wider public governance, structures and discriminations that kept the public from providing feedback in the first place and that shaped the extent of responsiveness by DHMTs to public feedback.

**Figure 3.**
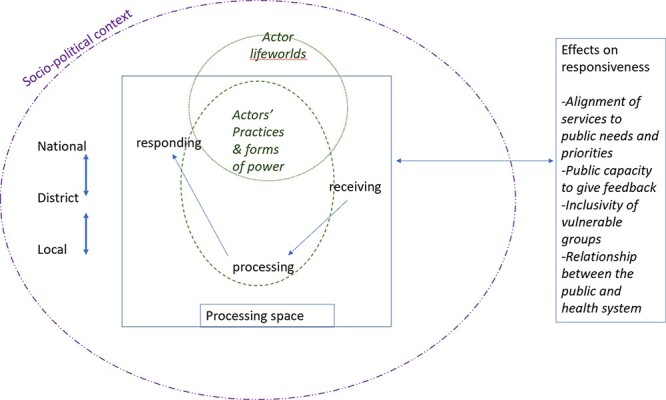
Conceptual framework illustrating how the exercise of power influences the functioning of spaces for receiving and responding to public feedback

Gaventa’s power cube and Long’s interface analysis were found to be complementary in analysis. The power cube supported the examination of the DHMT as a collective space and how this collective’s use of power was supported or constrained by structural factors. We found these structural factors to be related to the power cube’s levels of power and visible and invisible forms of power. For example, the national and regional levels of power commonly exercised visible power over DHMTs. Long’s actor interface analysis was useful in eliciting where and with whom power lies within the DHMT and within the health system the DHMT is part of and why certain actions were taken (or not) by the DHMT concerning public feedback. Based on these findings, Figure 3 summarizes our ideas about the influence of power dynamics on district health managers’ actions in receiving and responding to public feedback.

This framework illustrates how structural influences and the agency of actors interplay within the spaces where decision-making about public feedback happens. It suggests that actors’ life-worlds are shaped by the contexts in which they find themselves. These in turn shape the actors’ power practices and forms of power in receiving and responding to public feedback. Within a processing space for public feedback such as the DHMT, power can be wielded in both positive and negative ways. How this power is exercised has a reinforcing effect on the public’s sharing of feedback. Positive power practices support the generation of responses and even more feedback from the public. Negative power practices could limit the generation of responses and the public’s sharing of feedback or prevent the public from building claimed spaces. However, causation is not linear as actor interfaces form and re-form resulting in power struggles, the effect of which could be to support or undermine the practice of responsiveness, including by excluding the voices of marginalized groups. Furthermore, in these power struggles, power may flow bottom-up, contrasting with the traditional top-down flow, particularly where the public reacts to domination. These findings are relevant to HPSR investigators with an interest in health system responsiveness. They could, for example, build on this article and extend the framework presented in Figure 3 with research that considers experiences in other types of spaces such as HFCs or public participation forums where public feedback is received and responded to.

Our findings suggest that responsiveness might be strengthened by recognizing and building on actor life-worlds, while paying attention to the broader context in which the life-worlds are embedded. For example, politicians were observed to dominate the public and DHMTs, a power practice underpinned by the personal concerns of advancing political careers. In decentralized contexts such as Kenya (Nyikuri *et al.*, 2017; Tsofa *et al.*, 2017; McCollum *et al.*, 2018) and Uganda (Kapiriri *et al.*, 2003; Razavi *et al.*, 2019; Boydell *et al.*, 2020), the critical resource allocation and decision-making roles of political actors appeared to enhance this practice. Thus, leveraging the personal concerns of politicians (such as the interest to appeal to their voter base) in such contexts could deepen the practice of responsiveness to public feedback. The importance of recognizing the influence of political power in supporting policy implementation has been demonstrated in other published literature (Dalglish *et al.*, 2015) although this study reported findings of a highly centralized political context. These findings are of value to health managers, particularly those who interact with political appointees and elected political representatives, as they draw attention to the need to appreciate the motivations of political actors who influence health system resourcing, planning and implementation.

Leveraging politicians’ personal concerns requires careful application. This is because such an approach could direct responsiveness away from vulnerable groups (who often do not form a large voter base), thus undermining the equity goal of responsiveness. To address this challenge requires lowering the costs of participation in feedback channels. Health managers in collaboration with CSOs can lower participation costs by developing interventions aimed at off-setting invisible power by building the agency of the public, particularly vulnerable groups. Specific actions include increasing information available to the public regarding how their voices can be heard and supporting the public to present their concerns. In the reviewed articles, these activities were mainly conducted by NGOs, which raised the public’s awareness about their rights and supported participatory platforms where citizens engaged with duty bearers at the community, health facility and district levels (Butler *et al.*, 2020; Blake *et al.*, 2016; George *et al.*, 2018; Boydell *et al.*, 2020; Butler *et al.*, 2020). DHMTs can also participate in efforts to share power with the public by strengthening feedback channels, particularly those within the DHMT’s mandate such as HFCs or VHTs. Such efforts could include vigilance to ensure that invited spaces are truly inclusive (including marginalized groups) and support participants’ effective involvement. Specific actions here include, for example, providing timely information on invitations to public participation meetings and on-going rather than one-off engagement of the public (Shayo *et al.*, 2012).

DHMTs’ life-worlds related to their managerial positions of authority can also be leveraged to strengthen responsiveness. The study findings suggest that collaborative practices appear to hold promise for building responsive systems. Efforts by NGOs and research teams therefore need to support DHMTs to receive and respond to feedback, rather than working in parallel. Where these processes occur through a feedback channel not supported by the public health system, there needs to be a link back to the DHMT and public health system decision-makers. Such an approach could support learning and system-wide change. In the reviewed papers, where NGOs worked to strengthen pre-existing channels with the participation of DHMTs, there seemed to be increased trust in health system agents and improvements at the system level (Blake *et al.*, 2016; George *et al.*, 2018; Boydell *et al.*, 2020; Butler *et al.*, 2020). However, where NGOs operated independent of the DHMT and evolved their feedback mechanisms, such as in the study by Jacobs and Baez Camargo (2020), there was little reported improvement in public trust in district-level actors, and hardly any public feedback reached the DHMT.

Observations of organizational hierarchies in the reviewed articles suggest that regional and national health managers have the power to influence DHMTs to be more responsive to public feedback. Drawing on top-down implementation theory (Hill and Hupe, 2002), regional- and national-level actors could align resources and organizational environments to support receiving and responding to feedback at the district level. They could also hold DHMTs accountable for weak or no handling of public feedback. However, hierarchical power would need to be exercised to provide a supportive environment rather than demanding compliance. Literature cautions that multiple demands for compliance push managers to prioritize certain courses of action over others and that this could undermine responsiveness to the public (Nxumalo *et al.*, 2018). In this review, many DHMTs experienced constraints on their flexibility to act due to guidelines and requirements for vertical performance accountability. To guard against this, emphasizing responsiveness to the public combined with transparency about actions taken in response to feedback and autonomy in decision-making is likely to contribute to orienting DHMTs outwards to the public and therefore to building responsiveness.

Another way to strengthen responsiveness could include efforts targeting at DHMTs world-views. In the studies reviewed, we identified mindsets among DHMT members such as little value for public feedback. Literature on strengthening district-level leadership and management suggests that setting up platforms for reflection and supportive supervision among DHMTs has the potential to shape mindsets about the value and legitimacy of public participation in health system decision-making (Cleary *et al.*, 2017; Nzinga *et al.*, 2021). Reflective practice can yield positive results in improving leadership and individual and team behaviours (Cleary *et al.*, 2017; Nzinga *et al.*, 2021). However, for reflective practice to have these effects, certain organizational conditions need to be in place that allows individual and group reflective practices to trigger organizational change (Nicolini *et al.*, 2003). Nicolini et al. (2003) suggest that such an organizational change is possible even in highly fragmented and politicized organizations if the reflective practice is participatory and has the support or authorization of higher system levels (Nicolini *et al.*, 2003). In LMIC contexts, this would include the support of regional- and national-level bureaucrats.

This review has some limitations. By including only English-language articles, we excluded several studies from Lusophone and Francophone Africa and Latin America that might have offered insights into the study questions. The inclusion of only English language articles might also explain why a majority of papers reported on experience in African countries. While the majority of papers mentioned decentralized study settings, there were not enough contextual data to determine the form of decentralization that is whether deconcentration, delegation or devolution (Mills *et al.*, 1990) for all the studies. This paper is therefore limited in the extent to which it can draw conclusions on the differences in responsiveness across varying levels of decentralization. However, it is not unusual for syntheses to ‘work with an incomplete knowledge base’ (p 3) (Gilson, 2014) and, despite these limitations, our interpretive synthesis can provide a platform for future empirical work. Our synthesis work drew on a conceptual framework, which was both tested and adapted through this process. The adapted framework that we present therefore presents analytic generalizations of wider relevance.

## Conclusion

In adopting an interpretive synthesis approach and applying two complementary power lenses, this work has systematically identified the influence of social norms, structures and discrimination on power distribution among actors in the environment surrounding, and within, the DHMT in relation to health system responsiveness. Furthermore, our analysis of power has illustrated reactions to the use of power and non-traditional flows of power (beyond the commonly reported top-down flows of power from national to regional to local and then to individual). The review has also proposed a conceptual framework (Figure 3) that can be applied to consider how receiving and responding to public feedback plays out in other health system spaces. The findings emphasize the need for measures that recognize the varied life-worlds of the range of actors involved in receiving and responding to public feedback. Some of these measures include leveraging politicians’ power and personal interests while strengthening feedback channels to ensure meaningful public involvement and inclusivity, and interventions to shape DHMTs’ world-views and work environments to support responsiveness to public feedback.

## Supplementary Material

czac105_Supp

## Data Availability

The data underlying this article are available in the article and in its online supplementary material.

## Endnotes

1. The term DHMT is used to refer to these subnational health management teams in subsequent sections of the paper.

2. Community-Oriented Resource Persons (CORPs) is another term for community health workers used in the policy documents at the time. One CORP was expected to support 50 households in a community unit. CORPs were overseen by community health extension workers who are health professionals linked to primary care facilities (dispensaries and health centres) (O’Meara *et al.*, 2011).

3. Local Action Groups (LAGs) are a mechanism comprising multiple stakeholders operating within sub-districts and districts to complement the work of other health-related governance structures such as HFCs, multi-sectoral action teams and the community policy forum and to undertake local-level action for identified needs and priorities. The LAG roles were viewed as going beyond specific health facilities and/or a specific set of health issues to include broader social determinants of health (Cleary *et al.*, 2014).

4. This core team comprised the district hospital director, the deputy district hospital director and the head of the district financial department.

5. This region is the northern part of Kenya, which has historically had few resources allocated to it including during the colonial times.

6. Performance accountability refers to demonstrating and accounting for performance in light of agreed upon performance targets (Brinkerhoff, 2003, 2004; Mukinda *et al.*, 2020).

7. These other feedback mechanisms included VHTs (comprising community members who implemented community awareness and health education activities) and the charitable arm of the Buganda Kingdom in one district, which provided social services (including health) parallel to the public sector.

## Reflexivity statement

The group of authors includes four females and one male. The group comprises researchers from three countries, Kenya, South Africa and the UK with varying levels of experience and seniority in health systems and policy research. Two members of the authorship team are PhD students. The researchers’ areas of study are varied and include health system governance, and priority setting, analysis of health policy and its implementation in LMIC health systems, health services and qualitative methods.
