# Supplementary material for: How does power shape district health management team responsiveness to public feedback in low- and middle-income countries: an interpretive synthesis
Source: Health Policy Plan. 2022 Dec 6;38(4):528–51. doi: 10.1093/heapol/czac105 (PMC10089071; doi:10.1093/heapol/czac105)
Supplement: czac105_Supp [file czac105_supp.zip › Supplementary Material 1 ENTREQ statement reporting guideline for qualitative synthesis studies.docx]

Supplementary material 1: Checklist utilised in drafting manuscript drawn from Enhancing transparency in reporting the synthesis of qualitative research: the ENTREQ statement (Tong et al., 2012)

| No | Item | Guide and Description |
| --- | --- | --- |
| 1 | Aim | State the research question that the synthesis adresses |
| 2 | Synthesis & Methodology | Identify the synthesis methodology or theoretical framework which underpins the synthesis, and describe the rationale for choice of methodology |
| 3 | Approach to searching | Indicate whether the search was pre-planned or iterative |
| 4 | Inclusion criteria | Specify the inclusion/exclusion criteria (e.g. in terms of population, language, year limits, type of publication, study type) |
| 5 | Data sources | Describe the information sources used, and when the searches conducted the rationale for using the data sources |
| 6 | Electronic search strategy | Describe the literature search (e.g. provide electronic search strategies with population terms, and search limits |
| 7 | Study screening Methods | Describe the process of study screening and sifting |
| 8 | Study Characteristics | Present characteristics of the studies included |
| 9 | Study selection results | Identify the number of studies screened and provide reasons for study exclusion (e,g, for comprehensive searching, provide numbers of studies screened and reasons for exclusion) indicated in a flow chart |
| 10 | Rationale for appraisal | Describe the rationale and approach used to appraise the included studies or selected findings (e.g. assessment |
| 11 | Appraisal items | State the tools, frameworks and criteria used to appraise the studies or selected findings |
| 12 | Appraisal process | Indicate whether appraisal was conducted independently by more than one reviewer and if consensus was required |
| 13 | Appraisal results | Present results of the quality assessment and indicate which articles if any were weighted/excluded based on the assessment and give the rationale |
| 14 | Data Extraction | Indicate which sections of the primary study were analysed and how the data were extracted from the primary studies |
| 15 | Software | State the computer software used if any |
| 16 | Number of reviewers | Identify who was involved in coding and analysis |
| 17 | Coding | Describe the processes of coding *(e.g. line by line coding* |
| 18 | Study comparison | Describe how comparisons were made within and across studies |
| 19 | Derivation of themes | Explain whether the process of derivation of themes was inductive or deductive |
| 20 | Quotations | Provide quotations from the primary studies to illustrate themes/constructs and identify whether the quotations were participants’ quotations of the author’s interpretation |
| 21 | Synthesis output | Present rich compelling and useful results that go beyond a summary of the primary studies (e.g. *new interpretation, models of evidence, conceptual models, analytic framework, development of a new theory or construct*) |
|  |  |  |

TONG, A., FLEMMING, K., MCINNES, E., OLIVER, S. & CRAIG, J. 2012. Enhancing transparency in reporting the synthesis of qualitative research: ENTREQ. *BMC Medical Research Methodology,* 12**,** 181.
