# Supplementary material for: How does power shape district health management team responsiveness to public feedback in low- and middle-income countries: an interpretive synthesis
Source: Health Policy Plan. 2022 Dec 6;38(4):528–51. doi: 10.1093/heapol/czac105 (PMC10089071; doi:10.1093/heapol/czac105)
Supplement: czac105_Supp [file czac105_supp.zip › Supplementary material 2 Title and Abstract screening tool.docx]

# Supplementary Material 2: Title and Abstract screening tool

1. Does the citation (title or abstract) describe any aspect of District Health Management Team functioning or decision making?
   - - Yes,
     - No,
     - Can’t tell.
2. Does the citation (title or abstract) describe issues of public or community participation or inclusion in providing feedback to the health system at district-level?
   - - Yes,
     - No,
     - Can’t tell.
3. Does the citation (title or abstract) describe issues of power at district-level or among District Health Managers?
   - - Yes,
     - No,
     - Can’t tell

Reviewer Decision:

- If the reviewer answer is “No” to any of the questions, disqualify citation
- If the reviewer answer is “Yes” to all the questions, select article for retrieval /further screening and appraisal.
- If the reviewer answer is “Can’t tell” to one of the questions, select article for retrieval /further screening and appraisal.
