# Supplementary material for: How does power shape district health management team responsiveness to public feedback in low- and middle-income countries: an interpretive synthesis
Source: Health Policy Plan. 2022 Dec 6;38(4):528–51. doi: 10.1093/heapol/czac105 (PMC10089071; doi:10.1093/heapol/czac105)
Supplement: czac105_Supp [file czac105_supp.zip › Supplementary Material 4_Summary of articles and extracted content.docx]

Supplementary Material 4: Summary of reviewed articles and sample of extracted content

| **Author** | **Study objective** | **Geographical coverage** | **Mechanism/Channel through which public feedback can be/is conveyed** | **Content of feedback received** | **Details on processing feedback** | **Details on responding to feedback** |
| --- | --- | --- | --- | --- | --- | --- |
| **Studies describing health system functioning** | | | | | | |
| Razavi DS., Kapiriri L., Abelson J., Wilson M, 2019 | To examine district-level decision-makers’ perspectives on the  participation of different stakeholders, including challenges related to their participation | Three districts in Uganda | -Representation by political, cultural, traditional leaders | Feedback not specified | No details on processing feedback | No mention of response to feedback |
| Henriksson D.K., Peterson S.S., Waiswa P., Fredriksson M., 2019 | to investigate to what extent  district-specific evidence informed prioritisation of child  survival activities in the annual district work plans and  how stakeholders in the planning process perceived the  use of evidence. | Two districts in Uganda | -Community dialogue with caregivers of children under 5, Health providers and Village Health Teams | Feedback not specified | No details on processing feedback | No mention of response to feedback |
| Van Belle S., Mayhew S.H., 2016 | To assess governance arrangements and accountability practices of key health actors in a  Ghanaian health district to understand to what extent public accountability is achieved | Rural district in Ghana | -District health committee; sub-district health committee, and Community Health Planning Service committee; NGOs interacted with vulnerable groups (adolescents, women) | These mechanisms were not functioning, thus content of public feedback is not specified |  |  |
| Tuba M, Sandoy IF, Bloch P, Byskov J., 2010 | To assess local perceptions of fairness and legitimacy of  decision making related to the delivery of malaria services  at district level | Kapiri-Mposhi District, Zambia | Suggestion boxes, meetings between district health managers and community members, Community Health Workers, Neighbourhood Committees | -Uncaring and harsh attitudes by health providers,  -Long waiting time at the facilities,  -lack of responses to complaints made by the community members,  -Inadequate malaria medicines at health facilities and inadequacy of subsidized Insecticide Treated Nets, (including being required to pay more than the subsidized amounts by healthcare workers),  -Health worker absenteeism | -Some feedback was not acknowledged by district health managers for example complaints about health provider behaviour towards health service users at facility level and complaints about waiting time | -In response to other feedback for example over-pricing of subsidized ITNs, district health managers in collaboration with NGOs supplying the ITNS set up a monitoring system to track number and price of ITNs |
| O’Meara WP, Tsofa B, Molyneux S, Goodman C, 2011 | To examine implementation of national planning guidelines including the engagement of communities in health sector priority setting | Kilifi District, Kenya | As per guidelines, community dialogue in which community priorities would be collected by CORPs but these were instead shared by HFC members (community representatives) in practice | Community priorities included filariasis, skin infections, bilharzias, and chronic conditions such as hypertension, diabetes and arthritis, health issues affecting adults and the elderly, and substance abuse among the local youth | Identification of local priorities happened as a separate process from target setting and activity planning and budgeting. Due to the parallel nature of the two processes, only local priorities consistent with national indicators were included in district plans and budgets. | This resulted in service delivery activities that mainly matched national level priorities and not local priorities |
| Maluka S, 2011 | To analyse health care organisation and management systems, and  explore the potential and challenges of implementing Accountability for Reasonableness (A4R) approach to  priority setting in Tanzania. | Mbarali District, Mbeya Region,Tanzania | According to priority setting guidelines, health boards and health facility committees provide information on community priorities but this did not occur in practice, | No specific community priorities/public feedback reported on |  |  |
| Mukinda F.K., Van Belle S., George A., Schneider H., 2020 |  | Gert Sibande District, Mpumalanga Province, South Africa | District Health Council (includes political representatives), clinic health committees, National Advocacy organisation, Treatment Action Campaign; informal mechanisms through meetings. Majority of the accountability mechanisms emphasised performance accountability | No specific community priorities/public feedback reported on |  |  |
| Kapiriri L, Norheim OF, & Heggenhougen K, | To assess leaders’ & the public’s experiences with public participation in health  planning and priority setting at different levels within a  decentralized framework. | Nama Sub-county, Mukono district, Uganda:  Included respondents from national, district, sub-county, parish and village levels (village leaders & community members) | Health Unit Management Committees, local councils, hospital boards, public health committees, but these were reported not to be functioning well | No specific community priorities/public feedback reported on |  |  |
| Jacobs E & Camargo BC, 2020 |  | RRP, GBAO districts in Tajikstan, Central Asia | No formal voice mechanisms in either of the two study districts; but neighbour committees & NGO supported CBOs were the main channels for feedback but these did not link back to the district health system; community members evolved informal mechanisms with health providers at facility level | Informal payments at district hospitals  -General public distrust in the health system, but more trust reported in the lower peripheral facility levels |  |  |
| Parashar et al, 2020 | To analyse the role of actor relationships and power in the implementation of a free entitlement health policy | Himachal Pradesh, India | Specific community feedback mechanisms not identified |  |  |  |
| Nyikuri et al, 2017 | To describe how district managers experienced  and interpreted this change within a context of a  rapidly devolving health system in Kenya | Coastal county in Kenya | Views of the community were to be collected through the community unit and shared upwards to the facility and health system levels | Content of community feedback not described |  |  |
| McCollum et al, 2018 | To provide a power analysis of priority-setting at county level in Kenya, following devolution | Multiple counties in Kenya | Public participation forums but these are poorly attended | Content on community feedback was not reported |  |  |
| Tsofa et al, 2017 | To examine the early effects of devolution  in Kenya on health sector planning, budgeting and  financial management. | Kilifi County in Kenya | Public participation initiated by County Treasury | Content on community feedback was not reported |  |  |
| Cleary et al, 2014 |  | South Africa | Local Action Groups, Health Facility Committees, |  |  |  |
| **Intervention studies** | | | | | | |
| Butler et al, 2020 | To contribute to the evidence base by providing lessons from a strategic, multitool, multi-level social accountability project | 5 districts, Malawi | CSOs, bwalo forum (community dialogue) at community and district level, radio listening clubs | -poor referral systems and lack of emergency transport equipment and systems; inadequate staff and attendance at health centers and negligent or unfriendly workers; lack of ‘youth friendly’ health services, clinics for children under five, and functional  maternity wards; shortages of drugs and supplies  and the suspicion that health workers divert or sell ‘free’  drugs; lack of electricity or adequate space in health centers; poor water and sanitation in health facilities; issues related to traditional customs and beliefs (e.g. child marriage, home deliveries); gender-based violence and lack  of male involvement in RMNCAH; and, lack of health  budget experience and training for newly appointed  councillors. | Most structural issues were passed on to the national level for action. These required district health managers to travel to the capital | -Authors suggest some community level action was taken but not clear what responses were implemented at community level. However structural issues (e.g. staff shortages, drug thefts and stockouts,  a weak referral system and inadequate infrastructure) reportedly took time to be fixed. |
| Blake et al, 2016 | To assess the effectiveness of engaging multiple health and non-health sector stakeholders  to improve MNH services at facility level | Ashanti and Volta regions, Ghana | Facility score-cards. A multi-disciplinary team scored facilities by assessing the health facilities’ environment to provide emergency obstetric services, assessing client satisfaction with services | Staffing shortages, availability of drugs, availability of equipment, accessibility challenges to health facilities | Analysis of score-card results shared at district, health facility & community level meetings | Community leaders identified actions that could be taken at community level, e.g. fund-raising to improve roads, to buy an ambulance. Between two assessments, five facilities obtained an emergency vehicles/ambulance—either through the purchase of a new  vehicle or through an improved referral system using the existing  district hospital vehicle. |
| Boydell et al, 2018 | To examine how changes are produced in a social accountability project (The Health Accountability Project) and what happens in the implementation process | Three districts in Central Uganda | CSOs undertook budget analyses of FP/RH spending and prepared a brief on local performance, mapped the district decision-making, created community groups, and facilitated dialogues with decision-makers. The CSOs worked with Village Health Teams comprising Community Health Workers and in one district with the charitable arm of the Buganda Kingdom (traditional kingdom) which provides social services | -Requests for outreach services for FP  -Threat of violence from male partners because of contraceptive use  - | No specific mention of specific processing of feedback data, rather for some feedback action was taken as implementation was ongoing | increased budget allocations for FP/RH services in two districts, increased staffing levels, and the development of an operating theatre in one district. Increased number of delivery beds in participating villages and included, and increased mobile services and blood donations; consultations with communities on health matters during district planning  -Increased uptake & demand for FP services |
| George et al, 2018 | To examine how community action can improve care seeking and  service delivery of maternity services for marginalized  communities, | Gujarat, India | -Community report cards from 2395 women's self-reported receipt of information on entitlements and use of  services over 3 years of implementation monitored prospectively through household visits); Women were engaged through their community platforms-women's collectives, self-help groups, village development committees and dairy co-operatives to create awareness on entitlement, and later collect feedback; hotline for women to call in case of obstetric emergencies | -Request for resumption of services suspended in several facilities  -Few outreach clinics in hard to reach areas  -low care seeking among women from vulnerable groups (e.g. higher numbers of home deliveries)  -higher use of private facilities among vulnerable women | Data collected by volunteers were sent to the supporting NGO staff who collated data from the monitoring tool into the report cards. A color-coded system was developed to denote whether levels of service receipt were  poor (red), average (yellow) or good (green).  -NGO staff would then lead the dialogue with health providers and health managers | -Increased awareness among marginalized women about health entitlements related to their health needs and rights; Restarting  of services (increasing the number of outreach clinics in hard to reach areas; Initiating deliveries in a previously defunct facility), repairs that improved the quality of the service environment (fixing leaks and toilets), better relationships  between community members and government providers (health trainings by government providers for women's collectives, invitation to NGO partners to attend  block level maternal death review meetings), and addressing  inappropriate practices (kick-backs between female community level providers and private providers, private hospital not providing services as per the public-private  insurance scheme) |
| Zulu J.M., Michelo C., Msoni C., Hurtig A.-K., Byskov J., Blystad A., 2014 | To examine local perceptions and practices related  to what was perceived as ‘fair’ priority setting (baseline  study) and the potential evolvement of such perceptions  and practices over time as a result of an AFR  based intervention (evaluation study) | Kapiri-Mposhi District in Zambia | Neighbourhood Committees | No content of feedback specified, but notes that there was little input by the community during priority setting |  |  |
| Byskov et al, 2014 | To assess  knowledge about the relevance and usefulness of the (Accountability for Reasonableness)  AFR concept as well as about the implementation  process and potential outcomes from diverse contexts. | Malindi district in Kenya, Kapiri-Mposhi district in Zambia, Mbarali district in Tanzania | In Tanzania-Meetings at community level between district health managers and community members  -In Kapiri-Mposhi, Zambia-neighbourhood health committees  community meetings,  suggestion boxes, and  development committees | Identified community priorities in Tanzania included-requestsfor construction of new health facilities, solving problems with procurement of drugs, supplies, and equipment, and shortage of health staff.  -Content of community feedback from Kenya & Zambia not identified | - After priorities across the health system (starting from the community level) were consolidated at district level, they were disseminated again to the public to provide opportunity for appeal prior to approval and on-ward submission to regional level  -In Malindi, Kenya, priorities were publicized through a newsletter and posting of adopted district priorities at in facility notice boards  -In Kapiri Mposhi, Zambia, the DHMT increased its  use of existing ways to make decisions and reasons public  to the community. This included the use of drama groups, neighbourhood health committees, traditional  birth attendants, posters, community meetings, information sessions at the clinics, and the development committees. | -The AFR intervention was most fully implemented in Zambia, where outcomes included improvements in identification of local priorities, and greater involvement of stakeholders in priority setting. However, the project duration in all three countries was too short to demonstrate  effects in terms of changes in the ultimate  outcomes of AFR for quality, equity, and trust and for  health outcomes |
| Maluka et al, 2011 | To evaluate the experiences of implementing the AFR approach in Mbarali District,  Tanzania, in order to find out how the innovation was shaped, enabled, and constrained by the interaction between contexts, mechanisms and outcomes | Mbarali District, Tanzania | CHMT members travelled twelve villages in the district to solicit priorities from the community; | Content of community feedback not described | Priorities collated at the district level were disseminated to the public and facilities prior to submission to regional level to provide an opportunity for appeal. These priorities were pinned on the notice board at the district hospital, district council offices, village council offices, ward executive offices, health centres, and dispensaries | No specific responses were identified |
