# Supplementary material for: How does power shape district health management team responsiveness to public feedback in low- and middle-income countries: an interpretive synthesis
Source: Health Policy Plan. 2022 Dec 6;38(4):528–51. doi: 10.1093/heapol/czac105 (PMC10089071; doi:10.1093/heapol/czac105)
Supplement: czac105_Supp [file czac105_supp.zip › Supplementary Material 5_Summary of content of feedback.docx]

Supplementary Material 5: Summary of content of public feedback from reviewed articles

| Provider-client relations | Infrastructure, staffing and commodity-related issues | Requests for introduction of new services & challenges in accessing services | Other issues not directly related to health service delivery that impacted uptake of health services |
| --- | --- | --- | --- |
| -uncaring and harsh attitudes by health providers (Tuba et al., 2010)  -unwelcoming reception approaches (such as neglecting the principle of first-come first-serve and emergencies first)(Tuba et al., 2010)  -politicians being prioritised for services at facilities(Tuba et al., 2010)  -lack of responses to complaints made by the community members,  -health worker absenteeism(Tuba et al., 2010)  -Suspicions that HCWs divert drugs(Butler et al., 2020) | -inadequate malaria medicines at health facilities and inadequacy of subsidized Insecticide Treated Nets, (including being required to pay more than the subsidized amounts by healthcare workers)(Tuba et al., 2010)  -poor referral systems and lack of emergency transport equipment and systems(Butler et al., 2020, Blake et al., 2016)  -lack of clinics for children under five, and functional maternity wards(Butler et al., 2020) (Butler et al, 2020)  -shortages of drugs and supplies(Butler et al., 2020, Blake et al., 2016)  - inadequate FP/RH* supplies and commodities(Boydell et al., 2020)  - poor accessibility of some facilities (due to bad roads)(Blake et al., 2016)  -inadequate staffing of maternity and FP areas(Butler et al., 2020, Blake et al., 2016, Boydell et al., 2020) | -lack of ‘youth friendly’ health services(Boydell et al., 2020)  -Inadequate staffing for Family Planning (Boydell et al., 2020)  -Requests for inclusion of filariasis, skin infections, bilharzias, and chronic conditions such as hypertension, diabetes and arthritis, health issues affecting adults and the elderly, and substance abuse among the local youth in district priorities (O'Meara et al., 2011) | --poor water and sanitation in health facilities(Butler et al., 2020, Blake et al., 2016)  -issues related to traditional customs and beliefs (e.g. child marriage, home deliveries)(Butler et al., 2020)  -gender-based violence and lack of male involvement in RMNCH(Butler et al., 2020)  -lack of health budget experience and training for newly appointed councillors(Butler et al., 2020)  -socio-cultural norms that prevented access to FP/RH commodities(Boydell et al., 2020) |

Abbreviations: FP-Family Planning, RH-Reproductive Health, RMNCH-Reproductive Maternal Newborn and Child Health, HCW-Healthcare workers

BLAKE, C., ANNORBAH-SARPEI, N. A., BAILEY, C., ISMAILA, Y., DEGANUS, S., BOSOMPRAH, S., GALLI, F. & CLARK, S. 2016. Scorecards and social accountability for improved maternal and newborn health services: A pilot in the Ashanti and Volta regions of Ghana. *Int J Gynaecol Obstet,* 135**,** 372-379.

BOYDELL, V., NULU, N., HARDEE, K. & GAY, J. 2020. Implementing social accountability for contraceptive services: lessons from Uganda. *BMC Women's Health,* 20**,** 228.

BUTLER, N., JOHNSON, G., CHIWEZA, A., AUNG, K. M., QUINLEY, J., ROGERS, K. & BEDFORD, J. 2020. A strategic approach to social accountability: Bwalo forums within the reproductive maternal and child health accountability ecosystem in Malawi. *BMC Health Services Research,* 20**,** 568.

O'MEARA, W. P., TSOFA, B., MOLYNEUX, S., GOODMAN, C. & MCKENZIE, F. E. 2011. Community and facility-level engagement in planning and budgeting for the government health sector--a district perspective from Kenya. *Health policy (Amsterdam, Netherlands),* 99**,** 234-243.

TUBA, M., SANDOY, I. F., BLOCH, P. & BYSKOV, J. 2010. Fairness and legitimacy of decisions during delivery of malaria services and ITN interventions in zambia. *Malaria Journal,* 9**,** 309.
