# Supplementary material for: How does power shape district health management team responsiveness to public feedback in low- and middle-income countries: an interpretive synthesis
Source: Health Policy Plan. 2022 Dec 6;38(4):528–51. doi: 10.1093/heapol/czac105 (PMC10089071; doi:10.1093/heapol/czac105)
Supplement: czac105_Supp [file czac105_supp.zip › Supplementary material 3 Quality appraisal check.docx]

| Are the aims and objectives of the research clearly stated? Is the research design clearly specified and appropriate for the aims and objectives of the research? Do the researchers provide a clear account of the process by which their findings we reproduced? Do the researchers display enough data to support their interpretations and conclusions? Is the method of analysis appropriate and adequately explicated? |
| --- |

Supplementary material 3: Appraisal prompts for informing judgements about quality of papers (Dixon-Woods et al., 2006)

DIXON-WOODS, M., CAVERS, D., AGARWAL, S., ANNANDALE, E., ARTHUR, A., HARVEY, J., HSU, R., KATBAMNA, S., OLSEN, R., SMITH, L., RILEY, R. & SUTTON, A. J. 2006. Conducting a critical interpretive synthesis of the literature on access to healthcare by vulnerable groups. *BMC Medical Research Methodology,* 6**,** 35.
